# Correcting for artifactual correlation between misreported month of birth and attained height-for-age reduces but does not eliminate measured vulnerability to season of birth in poorer countries

**DOI:** 10.1093/ajcn/nqz111

**Published:** 2019-06-10

**Authors:** Amelia B Finaret, William A Masters

**Affiliations:** 1 Program in Global Health Studies, Allegheny College, Meadville, PA 16335, USA; 2 Friedman School of Nutrition Science and Policy, Tufts University, Boston, MA 02111, USA

**Keywords:** nutrition smoothing, height-for-age, measurement error, Demographic and Health Surveys, artifactual patterns, seasonality

## Abstract

**Background:**

Height-for-age z-scores (HAZ) are associated with month of birth (MOB) in many nutrition surveys, but that link could be an artifactual result of measurement error in child birthdates.

**Objective:**

We corrected estimates of the associations between HAZ and MOB for a common type of age misreporting, to measure the remaining seasonality in HAZ and identify country characteristics associated with vulnerability to seasonal changes in early life.

**Design:**

We used nationally representative repeated cross-sections from all available Demographic and Health Surveys (DHS), totaling 1,363,806 children from 218 surveys in 72 countries over 1986–2016, to estimate the seasonal patterns in HAZ by MOB within each survey. Then, we corrected these estimates for each survey's random errors in recorded birth month implied by differences in attained height between children reported as born in December of one year versus January of the next. Indicators of seasonal variation between other months were modeled as functions of national-level incomes using linear regression, and visualizations were constructed using nonparametric local polynomial smoothing regressions.

**Results:**

Over all surveys, misreporting MOB accounted for about one-eighth of the gap in attained height between the worst and best months to be born, which averaged 0.41 HAZ in the raw data and 0.34 HAZ after correction for age misreporting. A linear correction reduced apparent seasonality of HAZ by MOB in 49 of 72 countries, and the remaining nonartifactual differences by season of birth were larger in countries with lower average income per capita.

**Conclusions:**

Measurement error in child MOB helps to explain the association between attained height and seasonal variation in early life environments, but significant seasonality in HAZ by MOB remains in many poor countries. Higher national income is associated with smoother outcomes across birth months, and birth registration efforts would improve nutrition research.

## Introduction

Measuring the nutritional consequences of seasonal changes in disease exposure and the food environment can improve understanding of how poorer families cope with risk, avoid vulnerability, and gain resilience to the many nutritional risk factors they face (1, 2). Nutrition smoothing is the ability of an individual or a group of people to maintain stability in their nutritional status and health despite changes in their household and community environment, including seasonal fluctuations in weather as well as sudden shocks such as natural disasters or pest infestations (3).

Whether a population is able to smooth its nutritional outcomes can often be measured using associations between attained height and birth circumstances, due to the sensitivity of linear growth to different risk factors (4). Whatever a population's average level of stunting over the year, seasonal fluctuations can adversely affect children's human capital development (5) and linear growth (6–8). Direct measurement of relevant climate and weather variables might not be feasible, and in any case researchers can be interested primarily in whether attained heights are affected by all of the many factors that vary by season (9, 10). Seasonality in height and other nutrition indicators by month of birth (MOB) has been observed in several populations and varies depending on region (11). The mechanisms for these patterns might be related to the endocrine system (12), energy intake (13), dietary quality (14), poverty (15), birth- or conception-related factors (16), pregnancy-related factors (17), or the disease environment (18).

Analyses of seasonality in child heights depend on the accurate measurement of child birthdates (19, 20). This is a difficult task, especially because globally only ∼65% of children aged <5 y have had their births registered (21). Larsen et al. (20) recently discovered artifacts that cause an apparent seasonal pattern in child heights by MOB in the Demographic and Health Surveys (DHS). One of these artifacts is a gradient in height-for-age z-scores (HAZ), observed as an implausibly smooth linear increase in average HAZ monthly from January to December, and an implausibly large step change in mean HAZ from those reportedly born in December of one year to January of the next (19, 20). HAZs indicate linear growth for individual children compared with healthy children of the same age and sex. This implausible monthly gradient and December–January gap can be explained by random error in reported birth months.

Random measurement errors in exposure variables cause imprecise coefficient estimates, attenuation bias, and a loss of statistical power, but in this case random misreporting of birth months within calendar years interacts with linear growth to create artifactual seasonality in observed HAZ. The objective of this study was to correct for the implausible gradient in the relation between HAZ and MOB, and thereby reveal nonartifactual seasonality in attained heights by MOB across countries.

## Methods

We used a collection of nationally representative repeat cross-sections of data from the DHS to estimate patterns in child heights by MOB (22). The DHS are the largest collection of comparable health and nutrition microdata in the world, and are typically conducted at 5-y intervals in many low- and middle-income countries in collaboration with national statistics offices. Women of childbearing age (age 15–49 y) are the primary subjects of DHS data along with their children aged <5 y. Our focus was these children's height-for-age, for which we have about 1.4 million observations (**Supplemental Figure 1**). To build this dataset we appended 218 of the Standard DHS together in Stata 15/MP (23). This collection spanned 72 countries and 31 y. Then, we generated binary variables indicating reported MOB. Next, we estimated HAZ as a function of MOB, controlling for age-in-months and sex, by survey with mother fixed-effects using the ordinary least squares (OLS) estimator. Age and sex controls were used to improve the precision of estimated coefficients on MOB, reducing errors attributable to differences in the ages, sexes, or other attributes of children with each reported birth month in any given survey, or among siblings with the same mother in estimates that use maternal fixed-effects.

In Equation *1*, *i* indexes mothers, *j* indexes children, and *m* indexes months. *MoB_m_* denotes 11 binary indicators for reported MOB estimated with January as the omitted reference group, *Age* is measured as number of months in linear and quadratic terms, *Male* is a binary indicator which equals 1 if the child is male and 0 if the child is female, ${\delta _i}$ are mother fixed-effects, and ${\mu _{ij}}\ $is an independent and identically distributed error term. To account for correlation in omitted influences on HAZ in each survey site, robust SEs for each coefficient were estimated with clustering by enumeration area. Coefficient estimates from these models were used to construct indicators of seasonality in heights, using separate regressions for each survey.

(1) \begin{eqnarray*}
HA{Z_{ij}} &=& {\alpha _m}\ Mo{B_{mij}} + \ {\beta _1}Ag{e_{ij}}\ + \ {\beta _2}Age_{ij}^2 \nonumber \\
&& + \gamma Mal{e_{ij}} + {\delta _i} + {\mu _{ij}}
\end{eqnarray*}

To calculate and visualize gradients for worst-to-best months to be born, the matrix of estimated coefficients for the model in Equation *1*, by survey, was exported into a new database. Therefore, the set of results across surveys resulted in a matrix of estimated coefficients with 218 rows and 15 columns. The rows of the new matrix contained the sets of coefficient estimates by survey. The columns of the new matrix contained the coefficients estimated on the following variables: 11 month-of-birth binary variables, age, age^2^, sex, and a constant term. Also included were the following scalars in subsequent columns, by survey: *F* statistics, *R*^2^, and total number of observations from each OLS regression.

To correct the estimated coefficients on MOB for the measurement errors, we followed Larsen et al. (20) in assuming that the implausibly large gaps between December and January births and the implausibly smooth gradients observed in estimated coefficients over each successive month within the year were due to random misreporting of MOB. For children who were actually born in midyear (July), if their MOB is misreported earlier in the year (between January and June), then they are in fact younger than they are reported to be, and therefore their height-for-age would be calculated as lower than it should be, leading to lower average HAZs in the earlier months of the year. The reverse is true if their MOB is misreported as occurring later in the year (between August and December).

With equal probability that births in each month will be misreported as occurring in earlier or later months, the only children whose recorded birthdate is an unbiased estimate of their true age are those born at the midpoint of each year between June and July. To correct for the artifactual gradient in HAZ effects associated with each successive reported MOB, we subtracted one-twelfth of the estimated coefficient on December births from the estimate for each successive month, with one-half of the total added back so the gradient was rotated around the midpoint of each year and July 1 births became the reference category. Denoting the estimated coefficient on each recorded MOB as $\hat{\alpha }_m^s$, where *m* is 1 for January and 12 for December, the corrected estimate of true seasonal effects in each month net of measurement error was:

(2) \begin{eqnarray*}
Adj\hat{\alpha }_m^s = \ \hat{\alpha }_m^s - \left( {\frac{1}{{12}}\times m \times \hat{\alpha }_{12}^s} \right) + \ 0.5\left( {\hat{\alpha }_{12}^s} \right)
\end{eqnarray*}

This correction could be visualized as a linear rotation of each MOB coefficient around the midpoint of the year between June and July, allowing a comparison of true seasonality in HAZ with other indicators of health. For surveys with a larger estimated gap between December and January births ($\hat{\alpha }_{12}^s)$, the slope of this linear correction was steeper. Without the adjustment, seasonality in these other health outcomes and HAZ were not comparable, due to the systematic bias in estimated HAZ caused by random measurement error in birthdates. After we corrected the estimated coefficients for measurement error in child birthdates, we calculated 2 primary outcome variables to indicate seasonality in HAZ by survey, using the estimated coefficients on the MOB binary variables. Both seasonality indicators were calculated with the original raw coefficients and the corrected coefficients, to compare in visualizations. The first indicator of seasonality was the gap in HAZ between the worst-to-best months to be born in a given country and year (denoted *Gap*). This was calculated as the absolute value of the minimum of the estimated coefficients on MOB minus the maximum of the estimated coefficients on MOB (Equation *3*). This indicator reflected the potential disparity in HAZ between the worst and best months to be born for the participants of each given survey. The larger the estimated gap, the more substantial the presence of seasonality in heights.

(3) \begin{eqnarray*}
Ga{p_s} = \left| {Min\ \left\{ {Adj\hat{\alpha }_m^s} \right\} - Max\ \left\{ {Adj\hat{\alpha }_m^s} \right\}\ } \right|\
\end{eqnarray*}

(4) \begin{eqnarray*}
S{D_s} = SD{_m}\ \left\{ {Adj\hat{\alpha }_m^s} \right\}
\end{eqnarray*}

The second indicator of seasonality (Equation *4*) was the standard deviation of the corrected coefficients on *MoB_m_* (denoted *SD*). This indicator captured variation in vulnerability within the calendar year. The larger the estimated SD of coefficients on *MoB_m_*, the more substantial the presence of seasonality in HAZ. January was the reference month in estimated coefficients for each of these models, and July 1 was the corrected reference for the HAZ coefficients, so each coefficient could be interpreted as the mean difference in HAZ associated with each MOB, relative to others in their survey with the same age and sex, and relative to siblings with the same mother in regressions with maternal fixed-effects.

After the 2 seasonality indicators were constructed using the reduced 218-row matrix, the datasets of estimated coefficients were merged by DHS survey year with data on gross domestic product (GDP) per person, measured at purchasing power parity (PPP) prices, from the *Penn World Tables* (24). Missing values of GDP were linearly interpolated by year when possible. Then, we used OLS to estimate the relations between the 2 measures of seasonality, either *Gap* or *SD*, and GDP per person in that country at the time of the survey, as well as the survey year and year squared to capture any global trends over time (Equation *5*).

(5) \begin{eqnarray*}
Seasonalit{y_s} &=& {\alpha _s}\ + \ {\beta _1}{\rm{ln}}(GD{P_s}) + {\beta _2}Yea{r_s}\nonumber \\
&& + {\beta _3}Year_s^2 + {\epsilon _s}
\end{eqnarray*}

In addition to the regression analyses, we constructed visualizations of nutrition smoothing across months of birth before and after the adjustment for measurement errors and with respect to the national-level covariates. These visualizations were Epanechnikov-kernel weighted local polynomial smoothing regressions of degree zero. Using nonparametric methods is a flexible way to see differences across groups without having to assign a functional form. Nonparametric regressions estimated the means and CIs for each outcome as continuous functions of the variables on the *x*-axis: MOB, and the natural logarithm of GDP as an indicator of national-level incomes per person. We did not bring other data to the study for causal inference methods such as instrumental variables, or for replication and validation, because our aim was specifically to correct for the implausible gradient in HAZ over the calendar year that is observed on average globally. With additional data such as an instrumental variable that is correlated with true MOB and unrelated to HAZ, further research could identify causes of seasonality and modifiable factors to smooth nutrition outcomes at specific locations.

## Results

**Table 1** presents descriptive statistics for DHS by global region for Middle East/North Africa/West Asia/Europe, sub-Saharan Africa, South and Southeast Asia, Latin America and the Caribbean, Central Asia, and for the sample as a whole. The largest number of observations comes from sub-Saharan Africa, and HAZs were lowest in South and Southeast Asia with a mean of −1.48 SDs below the median.

**TABLE 1:** Descriptive statistics for HAZ by region for the collection of 218 DHS^1^

	Middle East and North Africa	Sub-Saharan Africa	Latin America and Caribbean	Central Asia	South and Southeast Asia	Total
Count	146,244	574,690	212,033	11,765	419,074	1,363,806
Mean	−0.84	−1.39	−1.08	−0.84	−1.48	−1.31
Median	−0.86	−1.41	−1.05	−0.84	−1.52	−1.32
SD	1.61	1.62	1.36	1.46	1.59	1.58
Minimum	−6.00	−6.00	−6.00	−5.97	−6.00	−6.00
Maximum	6.00	6.00	6.00	5.70	6.00	6.00

1 DHS, Demographic and Health Surveys; HAZ, height-for-age z-score.

**Table 2** presents descriptive statistics for the collapsed matrix of estimated coefficients across the 218 included surveys, and summarizes data on real GDP. The outcome variables are listed in order of how they were constructed, first correcting for measurement error in birthdates, then controlling for mother fixed-effects, and finally for measurement error in birthdates and mother fixed-effects. Across all surveys, there was a mean 0.23 HAZ points (0.17 SD) gap between reported December-born and reported January-born children. Before correcting for measurement error in child birthdates, the mean gap between the worst-to-best months to be born for HAZ was 0.41 HAZ, and after the linear correction, this mean gap declined to 0.34 HAZ. There were substantial country-level differences in these preadjustment and postadjustment means (**Tables 3** and **4**). After controlling for mother fixed-effects and clustering errors by community in the original survey-level regressions, the mean gap in HAZ decreased less, from 0.65 HAZ to 0.62 HAZ. Similarly, without controlling for mother fixed-effects, the mean of the SDs declined from 0.13 SD to 0.10 SD after accounting for measurement error in birthdates. After controlling for mother fixed-effects, the difference between premeasurement and postmeasurement error correction declined slightly, from 0.20 SD to 0.19 SD.

**TABLE 2:** Descriptive statistics for the collapsed dataset of estimated coefficients: outcome variables and GDPPC^1^

Variable	Description	Count	Mean	Median	Minimum	Maximum	SD
Gap_Raw	Gap between worst-to-best months to be born for child heights	218	0.41	0.38	0.09	1.04	0.15
SD_Raw	SD of estimated coefficients on months of birth as determinants of child HAZ	218	0.13	0.12	0.03	0.35	0.05
Dec-Jan Gap_Raw	Gap in HAZ between recorded December and January births	218	0.23	0.21	−0.14	0.91	0.17
Gap_Adj	Gap between worst-to-best months to be born for child heights, corrected for measurement error in birthdates	218	0.34	0.33	0.09	0.79	0.14
SD_Adj	SD of estimated coefficients on months of birth as determinants of child HAZ, corrected for measurement error in birthdates	218	0.10	0.10	0.03	0.24	0.04
Gap_FE	Gap between worst-to-best months to be born for child HAZ, with mother fixed-effects	218	0.65	0.56	—	2.39	0.37
SD_FE	SD of estimated coefficients on months of birth as determinants of child HAZ, with mother fixed-effects	217	0.20	0.18	0.04	0.67	0.11
Gap_Adj_FE	Gap between worst-to-best months to be born for child HAZ, corrected for measurement error in birthdates and mother fixed-effects	218	0.62	0.54	—	2.37	0.38
SD_Adj_FE	SD of estimated coefficients on months of birth as determinants of child HAZ, with mother fixed-effects, corrected for measurement error in birthdates	218	0.19	0.16	—	0.77	0.11
GDPPC	Expenditure-side real GDP at chained PPPs (in millions 2011US$), per capita	203	3187.96	2103.49	337.26	15,691.86	2840.05

1 GDP, Gross Domestic Product; GDPPC, Gross Domestic Product Per Capita; HAZ, height-for-age z-score; PPP, Purchasing Power Parity.

**TABLE 3:** Summary of the change in seasonality seen in child heights across MOBs by the gap between the worst and best months to be born in each country^1^

Region^2^	Country	Unadjusted gap	Corrected gap	Difference	Relative difference
S/SEA	India	0.357	0.222	0.136	−37.87
SSA	DR Congo	0.720	0.463	0.257	−35.65
S/SEA	Timor-Leste	0.672	0.433	0.239	−35.59
SSA	Chad	0.767	0.543	0.224	−29.24
S/SEA	Myanmar	0.491	0.361	0.130	−26.48
SSA	Angola	0.957	0.706	0.251	−26.23
SSA	Tanzania	0.492	0.368	0.124	−25.19
SSA	Gabon	0.626	0.473	0.153	−24.46
SSA	Zambia	0.545	0.423	0.122	−22.37
SSA	Comoros	1.325	1.115	0.211	−15.89
SSA	Mali	0.976	0.840	0.136	−13.94
MENA	Egypt	0.646	0.557	0.089	−13.76
SSA	Cameroon	0.659	0.571	0.088	−13.35
LAC	Haiti	0.619	0.541	0.077	−12.53
S/SEA	Pakistan	0.580	0.508	0.072	−12.34
LAC	Peru	0.268	0.235	0.033	−12.33
SSA	Madagascar	0.716	0.631	0.085	−11.87
SSA	Morocco	0.404	0.357	0.047	−11.56
SSA	Nigeria	0.505	0.449	0.056	−11.00
SSA	Liberia	0.650	0.579	0.071	−10.85
SSA	Swaziland	0.515	0.460	0.055	−10.68
SSA	Sierra Leone	0.826	0.743	0.084	−10.11
SSA	Togo	1.306	1.175	0.131	−10.06
SSA	Mozambique	0.516	0.466	0.049	−9.57
MENA	Yemen	0.520	0.473	0.047	−9.05
SSA	Guinea	0.786	0.720	0.065	−8.32
SSA	Burkina Faso	0.577	0.532	0.045	−7.76
SSA	Kenya	0.526	0.488	0.038	−7.23
SSA	Uganda	0.466	0.433	0.032	−6.95
S/SEA	Nepal	0.492	0.458	0.034	−6.87
SSA	Lesotho	1.036	0.965	0.071	−6.85
S/SEA	Sri Lanka	0.906	0.844	0.062	−6.84
S/SEA	Rwanda	0.449	0.422	0.026	−5.84
LAC	Brazil	0.439	0.415	0.024	−5.47
SSA	Malawi	0.480	0.454	0.026	−5.42
CA	Kyrgyz Republic	0.737	0.708	0.030	−4.00
S/SEA	Maldives	0.881	0.847	0.034	−3.86
CA	Tajikistan	0.585	0.567	0.018	−3.08
SSA	Senegal	0.994	0.964	0.030	−2.99
MENA	Armenia	0.887	0.862	0.025	−2.82
SSA	Benin	0.762	0.741	0.021	−2.79
SSA	Zimbabwe	0.730	0.711	0.019	−2.62
SSA	Niger	0.904	0.890	0.013	−1.49
SSA	Burundi	0.745	0.734	0.011	−1.48
SSA	Namibia	0.668	0.660	0.008	−1.24
LAC	Paraguay	0.454	0.449	0.005	−1.10
SSA	Central African Republic	0.734	0.731	0.002	−0.41
SSA	Republic of Congo	0.621	0.619	0.002	−0.32
SSA	Tunisia	0.982	0.980	0.002	−0.20
S/SEA	Bangladesh	0.578	0.579	−0.001	0.17
SSA	Gambia	0.738	0.741	−0.003	0.41
MENA	Turkey	0.545	0.559	−0.015	2.63
MENA	Albania	1.636	1.686	−0.050	3.06
LAC	Nicaragua	0.395	0.408	−0.014	3.42
S/SEA	Cambodia	0.524	0.546	−0.022	4.10
SSA	Ghana	1.065	1.126	−0.062	5.79
CA	Uzbekistan	1.473	1.559	−0.087	5.84
LAC	Colombia	0.519	0.551	−0.032	6.07
LAC	Trinidad and Tobago	0.507	0.539	−0.032	6.31
LAC	Dominican Republic	0.388	0.416	−0.028	7.22
MENA	Moldova	1.071	1.151	−0.080	7.47
SSA	Côte d'Ivoire	0.930	1.015	−0.086	9.21
LAC	Guatemala	0.367	0.403	−0.036	9.82
LAC	Bolivia	0.439	0.484	−0.045	10.34
S/SEA	Thailand	1.207	1.406	−0.199	16.49
SSA	Ethiopia	0.532	0.620	−0.088	16.55
LAC	Guyana	0.659	0.779	−0.120	18.21
MENA	Jordan	0.391	0.465	−0.075	19.10
SSA	São Tomé and Príncipe	0.452	0.553	−0.101	22.35
CA	Kazakhstan	0.948	1.161	−0.213	22.42
LAC	Honduras	0.204	0.299	−0.095	46.32
MENA	Azerbaijan	0.551	0.930	−0.379	68.78

1 Data shown are means of the *Gap* indicator of seasonality by included country (see text), before and after correcting for measurement error in child MOB. MOB, month of birth.

2 CA, Central Asia; LAC, Latin America and Caribbean; MENA, Middle East and North Africa; SSA, Sub-Saharan Africa; S/SEA, South/Southeast Asia.

**TABLE 4:** Summary of the change in seasonality seen in child heights across MOBs by the SD of coefficient estimates on MOBs in a multivariate regression^1^

Region^2^	Country	Unadjusted SD	Corrected SD	Difference	Relative difference
S/SEA	Myanmar	0.196	0.107	0.089	−45.41
S/SEA	Timor-Leste	0.213	0.133	0.080	−37.41
S/SEA	India	0.116	0.075	0.041	−35.34
SSA	Angola	0.280	0.187	0.093	−33.21
SSA	DR Congo	0.231	0.155	0.076	−32.90
SSA	Chad	0.243	0.169	0.074	−30.32
SSA	Tanzania	0.148	0.106	0.042	−28.42
SSA	Zambia	0.171	0.126	0.045	−26.46
SSA	Gabon	0.204	0.161	0.042	−20.88
SSA	Republic of Congo	0.201	0.160	0.042	−20.45
S/SEA	Pakistan	0.206	0.164	0.043	−20.44
MENA	Egypt	0.212	0.170	0.043	−20.01
LAC	Peru	0.086	0.069	0.017	−19.69
LAC	Haiti	0.188	0.152	0.037	−19.52
SSA	Mali	0.314	0.256	0.058	−18.54
SSA	Madagascar	0.231	0.190	0.041	−17.77
SSA	Malawi	0.154	0.127	0.027	−17.30
SSA	Sierra Leone	0.281	0.235	0.046	−16.22
SSA	Mozambique	0.173	0.146	0.027	−15.80
S/SEA	Nepal	0.152	0.129	0.022	−14.76
SSA	Morocco	0.135	0.116	0.019	−14.32
SSA	Kenya	0.169	0.148	0.022	−12.87
SSA	Liberia	0.219	0.191	0.028	−12.59
SSA	Swaziland	0.186	0.163	0.023	−12.37
SSA	Burkina Faso	0.180	0.158	0.022	−12.24
SSA	Comoros	0.365	0.323	0.042	−11.51
SSA	Guinea	0.242	0.218	0.024	−9.79
SSA	Lesotho	0.311	0.282	0.030	−9.42
SSA	Nigeria	0.152	0.138	0.014	−9.39
SSA	Togo	0.383	0.348	0.035	−9.13
CA	Kyrgyz Republic	0.273	0.249	0.025	−8.97
MENA	Yemen	0.152	0.138	0.014	−8.91
SSA	Uganda	0.148	0.135	0.013	−8.67
S/SEA	Rwanda	0.144	0.132	0.012	−8.46
S/SEA	Maldives	0.328	0.303	0.026	−7.62
MENA	Albania	0.457	0.424	0.033	−7.22
LAC	Paraguay	0.141	0.132	0.008	−6.38
S/SEA	Bangladesh	0.186	0.175	0.012	−6.26
S/SEA	Sri Lanka	0.276	0.260	0.015	−5.80
SSA	Burundi	0.220	0.208	0.013	−5.75
SSA	Senegal	0.299	0.287	0.012	−4.18
SSA	Zimbabwe	0.225	0.217	0.008	−3.56
LAC	Bolivia	0.138	0.133	0.004	−3.49
LAC	Nicaragua	0.134	0.131	0.003	−1.87
SSA	São Tomé and Príncipe	0.165	0.162	0.003	−1.82
CA	Tajikistan	0.166	0.163	0.003	−1.81
MENA	Moldova	0.303	0.299	0.003	−1.32
SSA	Benin	0.228	0.225	0.003	−1.21
LAC	Dominican Republic	0.116	0.115	0.002	−1.20
S/SEA	Thailand	0.363	0.361	0.002	−0.55
LAC	Colombia	0.160	0.160	0.000	0.10
SSA	Gambia	0.210	0.211	−0.001	0.48
LAC	Brazil	0.143	0.144	−0.001	0.70
MENA	Armenia	0.254	0.256	−0.002	0.79
LAC	Trinidad and Tobago	0.167	0.170	−0.003	1.80
SSA	Niger	0.249	0.256	−0.007	2.71
SSA	Central African Republic	0.241	0.249	−0.008	3.32
LAC	Guatemala	0.126	0.131	−0.004	3.70
MENA	Turkey	0.161	0.167	−0.007	3.93
SSA	Namibia	0.202	0.212	−0.010	4.70
SSA	Tunisia	0.292	0.306	−0.014	4.79
SSA	Cameroon	0.181	0.192	−0.011	5.89
S/SEA	Cambodia	0.155	0.166	−0.011	7.26
SSA	Ethiopia	0.167	0.182	−0.015	8.67
LAC	Guyana	0.233	0.254	−0.021	9.01
SSA	Côte d'Ivoire	0.281	0.308	−0.027	9.49
SSA	Ghana	0.330	0.361	−0.032	9.56
CA	Uzbekistan	0.405	0.451	−0.045	11.36
MENA	Jordan	0.119	0.135	−0.016	13.59
LAC	Honduras	0.075	0.087	−0.012	16.11
CA	Kazakhstan	0.306	0.385	−0.080	25.86
MENA	Azerbaijan	0.169	0.283	−0.114	67.46

1 Data shown are means of the *SD* indicator of seasonality by included country (see text), before and after correcting for measurement error in child MOB. MOB, month of birth.

2 CA, Central Asia; LAC, Latin America and Caribbean; MENA, Middle East and North Africa; SSA, Sub-Saharan Africa; S/SEA, South/Southeast Asia.

Tables 3 and 4 summarize the changes by country in 2 seasonality indicators before and after correcting for measurement error in child months of birth: *Gap* (Table 3) and *SD* (Table 4). Data in these tables were sorted by relative differences in each indicator, with the largest changes in seasonality after the linear correction at the top of the table and declining as the table continues. Seasonality in child heights in 49 countries declined by between −37.87% and −0.20% when measured by the gap between the worst and best months to be born. All regions of the world were represented in the 23 countries where seasonality measured by *Gap* increased from between 0.17% and 68.78%. Of the 10 countries in the DHS collection with the largest relative decrease in HAZ seasonality after the linear correction, 7 were located in sub-Saharan Africa (Table 3). When measured by the SD of coefficient estimates on months of birth, seasonality in child heights declined by between −0.55% and −45.41% in 50 of 72 countries after correcting for measurement error, and increased by between 0.10% and 67.46% for 22 countries.

**FIGURE 1:**
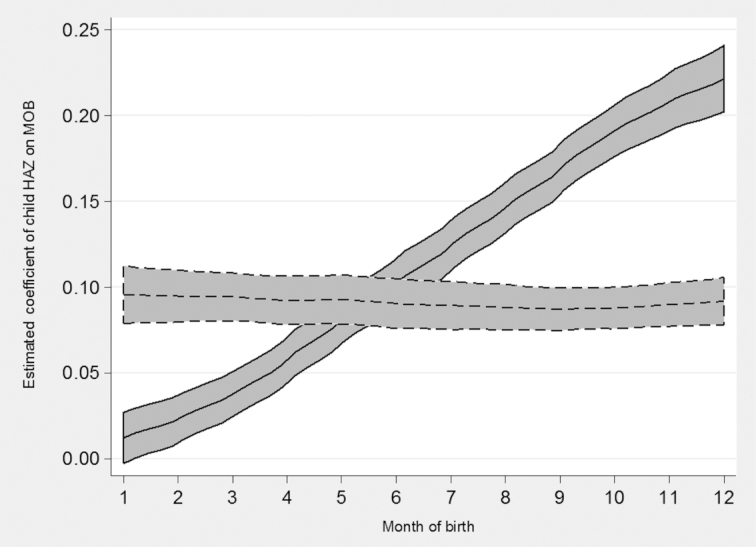
Estimated coefficients on child HAZ across 218 surveys. Lines are local polynomial smoothing regressions of degree zero with 95% CIs and an Epanechnikov kernel. Solid line is estimates before the linear correction for misreported MOB. Dotted line is after the linear correction for misreported MOB. HAZ, height-for-age z-score; MOB, month of birth.

**Figure 1** demonstrates the effects of the linear adjustment for measurement error in child MOB across the whole sample. The solid line shows the steady increase in estimated coefficients of HAZ on child MOB across the year, an artifactual relation that was the result of random measurement error in child MOB (19, 20). The dotted line shows how these estimated coefficients changed after correcting for the random measurement error, eliminating the implausibly large gap in HAZs between December-born and January-born children. Figure 1 includes all countries with available anthropometric data, and so a near-horizontal relation would be expected because weather and climate across the year vary greatly among the included countries (19, 20). Country-level investigations are necessary to ascertain where, if any, seasonality in child heights was still present after the adjustment.

**FIGURE 2:**
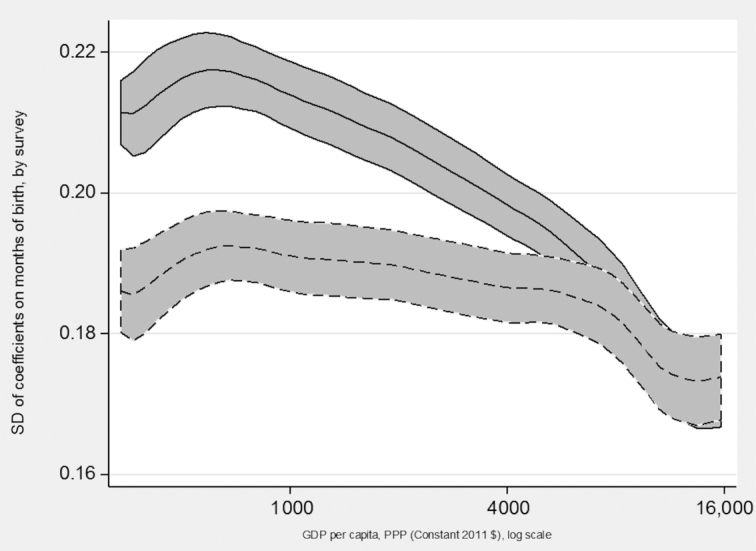
GDP and seasonality in HAZ across 218 DHS surveys: SD of estimated coefficients. Lines are local polynomial smoothing regressions of degree zero with 95% CIs and an Epanechnikov kernel. Solid line is estimates before the linear correction for misreported MOB. Dotted line is after the linear correction for misreported MOB. DHS, Demographic and Health Surveys; GDP, Gross Domestic Product; HAZ, height-for-age z-score; MOB, month of birth; PPP, Purchasing Power Parity.

**FIGURE 3:**
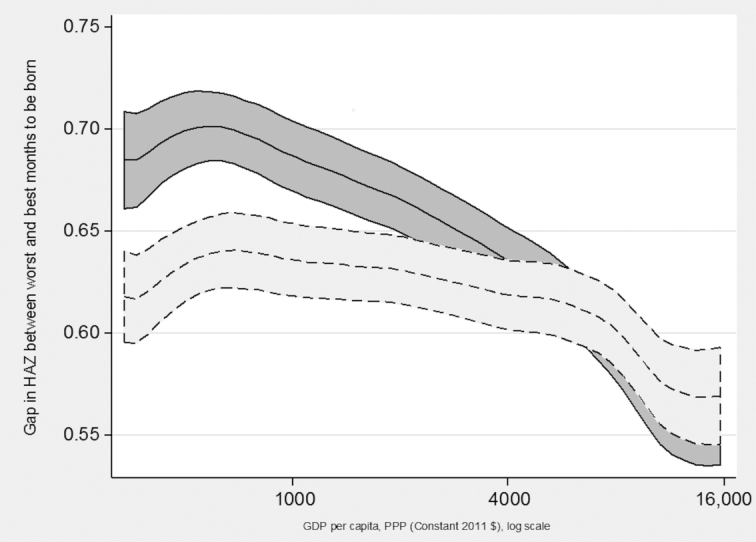
GDP and seasonality in HAZ across 218 DHS surveys: Gap between worst and best months to be born. Lines are local polynomial smoothing regressions of degree zero with 95% CIs and an Epanechnikov kernel. Solid line is estimates before the linear correction for misreported MOB. Dotted line is after the linear correction for misreported MOB. DHS, Demographic and Health Surveys; GDP, Gross Domestic Product; HAZ, height-for-age z-score; MOB, month of birth; PPP, Purchasing Power Parity.

**Figure 2** shows how seasonality in HAZ, measured by the gap between worst and best months to be born, was related to GDP per capita during the year of the survey in each country. A wider gap between the dotted and solid lines in these charts indicates more measurement error in MOB in the original DHS data. The solid line was not corrected for measurement error in birthdates, and showed a negative association between seasonality in HAZ and GDP per capita. This negative association was less pronounced but still present after correcting for measurement error in child birthdates, as indicated with the dotted line. The difference between preadjustment and postadjustment for measurement error in birthdates was nonexistent for the highest income countries, perhaps because the original measurement error was not especially pervasive for those surveys. For countries in the low-to-middle income range, the measurement error accounted for about 10% of the gap in HAZ between the worst and best months to be born. **Figure 3** shows a similar pattern for when seasonality was measured by the SD of MOB coefficients, where measurement error accounted for about 10% of the observed seasonality before the linear correction, but only for countries in the bottom and middle of the income distribution. For higher income countries, there were no differences preadjustment and postadjustment in the relation between HAZ seasonality and GDP per capita.

**FIGURE 4:**
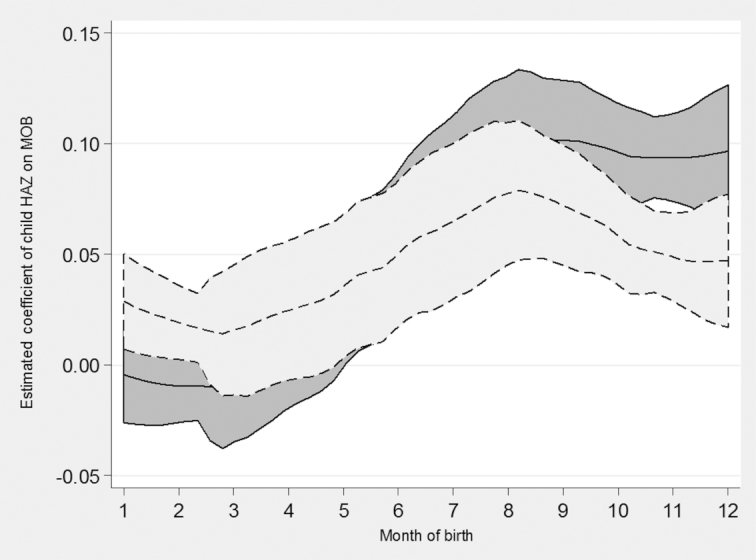
Estimated coefficients of child HAZ on MOB across 6 DHS in Bangladesh. Lines are local polynomial smoothing regressions of degree zero with 95% CIs and an Epanechnikov kernel. Solid line is estimates before the linear correction for misreported MOB. Dotted line is after the linear correction for misreported MOB. DHS, Demographic and Health Surveys; HAZ, height-for-age z-score; MOB, month of birth.

**FIGURE 5:**
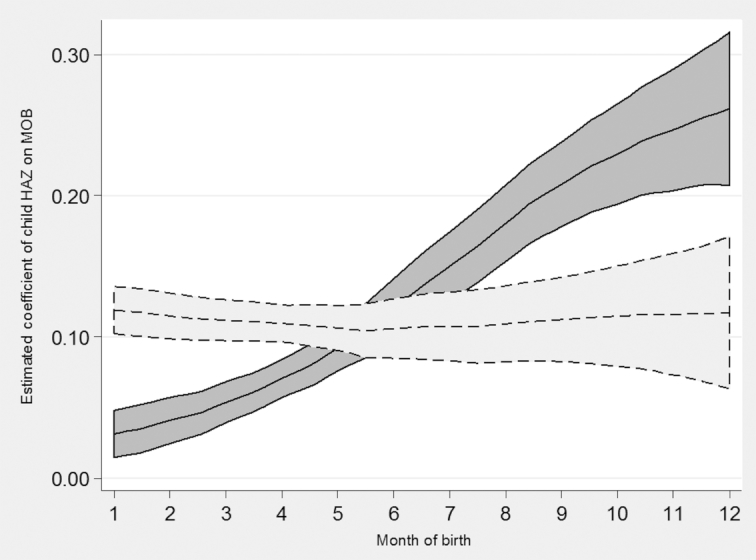
Estimated coefficients of child HAZ on MOB across 5 DHS in Zambia. Lines are local polynomial smoothing regressions of degree zero with 95% CIs and an Epanechnikov kernel. Solid line is estimates before the linear correction for misreported MOB. Dotted line is after the linear correction for misreported MOB. DHS, Demographic and Health Surveys; HAZ, height-for-age z-score; MOB, month of birth.

**Figures 4** and **5** are examples of country-level changes in the appearance of seasonality in HAZs before and after correcting for measurement error in child MOB. For illustration, we chose 2 countries, Zambia and Bangladesh, located in 2 different regions of the world, which had very different appearances of seasonality in child heights after the linear adjustment for measurement error in child MOB. First, directly comparing Figure 4 with Figure 5, seasonality in HAZs was still present in Bangladesh after correcting for measurement error in child MOB, but not in Zambia. In Zambia, any seasonality in HAZs was erased by the linear adjustment for measurement error in child MOB. Before correcting for the artifactual relation between HAZ and MOB, there was a gap of about 0.25 HAZ between December-born and January-born children in Zambia. After the adjustment, no gap was apparent between December- and January-reported births. In Bangladesh, there was still a gap of about 0.05 HAZ between the best month to be born (August) and the worst month to be born (March), even after correcting for measurement error in the MOBs. The comparison between Bangladesh and Zambia indicates that misreporting in MOB was more prevalent in Zambia than Bangladesh, perhaps reflecting the difference in birth registration systems between the countries.

The associations between seasonality in child HAZ and national income are presented **Table 5**. For illustration, an example of these regressions and the calculations for the *Gap* and *SD* seasonality indicators is presented in **Table 6** for Zambia, which had 5 Standard DHS surveys included in the full collection, implemented between 1992 and 2013. All models in Table 5 were estimated using the HAZ coefficients, corrected to account for measurement error in child birthdates, and the original regressions from which the coefficients came were estimated using mother fixed-effects. Seasonality in HAZs as measured by both indicators, *Gap* and *SD*, was negatively associated with GDP per capita. Given that the GDP covariate was log-transformed, the coefficients can be interpreted as semielasticities of HAZ seasonality with respect to GDP. Thus, a 1% increase in GDP at 2011 PPP prices was associated with a 0.065 reduction in the HAZ gap between the worst and best months to be born, and a 0.019 reduction in the SD across all estimated coefficients on months of birth. These estimated associations are meaningful because GDP typically grows over time and because seasonality indicators are measured at the population level. Although 0.065 HAZ points might not be clinically significant to an individual child, shifts in the seasonal distribution of HAZ of that magnitude are substantial. Seasonality in HAZ also declined over time, as indicated by the estimated coefficients on the time trend in columns 3 and 4 of Table 5. Each additional year reduced the HAZ gap between the worst and best months to be born by 0.01 HAZ points on average, and reduced the SD across all estimated coefficients on months of birth by 0.003 on average. Income and time are colinear due to economic development during this period, and the closest correlation between seasonality in HAZ and income is shown in the models in columns 5 and 6. In summary, seasonality in HAZ decreased slowly over time and has a small negative association with GDP, after correcting all estimates for measurement error in MOB. Low *R*^2^ values for the models in Table 5 are likely due to the relatively coarse measurement of population well-being in the GDP indicator. Several other household- and individual-specific factors also affect seasonality in HAZ, such as care practices, food intake, disease status, and livelihoods. By design, the models presented in Table 5 were not intended to account for most of the seasonality in HAZ, only to assess the associations between GDP and seasonality in HAZ. We would not expect GDP and time to be the sole determinants of seasonality in HAZ at the national level, but data limitations and the potential for measurement error precluded the use of other possibly relevant variables.

**TABLE 5:** Associations between seasonality in HAZ and GDP^1^

Variable	(1) HAZ *Gap*	(2) HAZ *SD*	(3) HAZ *Gap*	(4) HAZ *SD*	(5) HAZ *Gap*	(6) HAZ *SD*
Log(GDP)	−0.0646**	−0.0185***	—	—	−0.0655***	−0.0189***
	(*P* < 0.001)	(*P* < 0.001)			(*P* < 0.001)	(*P* < 0.001)
Year	—	—	−0.0097**	−0.00273**	−2.489	−0.862
			(*P* = 0.001)	(*P* = 0.002)	(*P* = 0.122)	(*P* = 0.072)
Year, quadratic	—	—	—	—	0.000619	0.000215
					(*P* = 0.123)	(*P* = 0.073)
Constant	1.292***	0.380***	20.01***	5.658**	2501.7	865.2
	(*P* < 0.001)	(*P* < 0.001)	(*P* = 0.001)	(*P* = 0.001)	(*P* = 0.120)	(*P* = 0.071)
*n*	203	203	218	218	203	203
*R* ^2^	0.065	0.061	0.048	0.044	0.125	0.120

1 Numbers in column headings are model numbers. Coefficients are OLS estimates of the associations between each given variable and an indicator of seasonality: the absolute value of the gap between the worst and best months to be born (*Gap*) and the SD of estimate coefficients on months of birth (*SD*), after correcting for measurement error in month of birth. Covariates are measured at national and annual levels. Original models were estimated by survey for 218 surveys using OLS for HAZ as a function of age, age^2^, sex, and mother fixed-effects. **P < 0.01, ***P < 0.001. GDP, Real 2011 Gross Domestic Product at Purchasing Power Parity; HAZ, height-for-age z-score; OLS, ordinary least squares

**TABLE 6:** Example calculations correcting for measurement error in child birthdates: 5 DHS in Zambia^1^

	Uncorrected estimates					Corrected estimates				
Survey year	1992	1996	2001	2007	2013	1992	1996	2001	2007	2013
Constructed indicators of seasonality										
Gap between worst-and-best months (Gap)	0.782	0.404	0.682	0.372	0.483	0.615	0.278	0.384	0.533	0.304
SD across month of birth coefficients (SD)	0.221	0.131	0.200	0.121	0.181	0.174	0.083	0.118	0.140	0.113
December–January Gap	0.293	0.316	0.182	0.419	0.346	0.119	0.129	0.074	0.171	0.141
Estimated coefficients on original OLS regressions										
February reported birth	−0.143	0.065	−0.178	0.183	0.018	−0.059	0.139	−0.018	0.360	0.117
March reported birth	−0.112	−0.038	−0.004	0.341	−0.171	−0.052	0.015	0.111	0.467	−0.100
April reported birth	−0.263	−0.072	0.003	0.555	−0.168	−0.227	−0.040	0.072	0.631	−0.125
May reported birth	−0.519	0.181	−0.122	0.260	−0.086	−0.507	0.192	−0.099	0.285	−0.072
June reported birth	−0.165	−0.010	−0.028	0.328	0.124	−0.177	−0.021	−0.051	0.303	0.110
July reported birth	−0.169	0.109	0.165	0.406	0.158	−0.205	0.078	0.096	0.330	0.115
August reported birth	−0.096	0.173	−0.063	0.501	0.246	−0.156	0.121	−0.177	0.375	0.175
September reported birth	0.007	0.216	0.171	0.346	0.278	−0.077	0.143	0.011	0.169	0.179
October reported birth	0.078	0.332	0.277	0.500	0.294	−0.030	0.238	0.071	0.273	0.166
November reported birth	0.220	0.259	0.194	0.375	0.128	0.089	0.144	−0.058	0.098	−0.028
December reported birth	0.263	0.231	0.504	0.555	0.312	0.108	0.095	0.206	0.227	0.128
Age, mo	−0.086	−0.101	−0.114	−0.095	−0.084	—	—	—	—	—
Age in months, squared	0.001	0.001	0.002	0.001	0.001	—	—	—	—	—
Child is male	−0.175	−0.036	−0.137	−0.136	−0.146	—	—	—	—	—
Constant	−0.466	−0.547	−0.389	−0.510	−0.345	—	—	—	—	—
*n*	4905	5503	5430	5096	11,373	—	—	—	—	—
*R* ^2^	0.266	0.241	0.295	0.173	0.142	—	—	—	—	—
*F*-statistic	34.110	29.040	40.800	18.210	30.990	—	—	—	—	—

1 The lefthand 5 columns are uncorrected for the linear gradient in the relation between HAZ and MOB. The righthand 5 columns have had a linear correction for this artifact. DHS, Demographic and Health Surveys; HAZ, height-for-age z-score; MOB, month of birth; OLS, ordinary least squares.

## Discussion

Even after accounting for random measurement error of birthdates that leads to spurious patterns in child heights throughout the year, seasonality in HAZ by MOB was still present in many of the poorest countries. This indicates that season of birth is still a determinant of linear growth in many but not all contexts, threatening long-term human capital development. Many of the countries with remaining nonartifactual seasonality in HAZ are in sub-Saharan Africa. In 9 countries—Côte d'Ivoire, Comoros, Ghana, Moldova, Kazakhstan, Togo, Thailand, Uzbekistan, and Albania—the remaining nonartifactual gap in HAZ between the worst and best months to be born was still >1.0 HAZ.

Country-specific seasonal patterns can be helpful for interpreting these results, for example, in Bangladesh. The main lean season in Bangladesh occurs during October–November (13, 25). Given the patterns seen in HAZ by MOB in Bangladesh after correcting for measurement error in child MOB, it appears that being born during the rice harvest season in February–March is worse for future height attainment than being born 2 months before the lean season in October–November. Therefore, having the complementary feeding stage begin in August, right before the lean season, is worse for subsequent linear growth compared with being born just before the lean season when newborn infants would be protected from food shortages by breastfeeding, and then be able to start their complementary feeding stage as the harvest season begins. Further location-specific analyses are important to understand specific issues related to birth registration systems and other constraints on national health survey accuracy.

The negative association between overall seasonality in child heights (after correcting for misreported MOB) and national-level incomes reflects height as a cumulative, intergenerational indicator of well-being. The negative association between seasonality in heights and GDP also speaks to the broad range of policies and conditions needed to promote resilience and protect families from adverse conditions throughout the calendar year. Further work is necessary to understand the determinants of nutrition smoothing in specific contexts, including the strategic use of longitudinal data, the concurrent measurement of agriculture, nutrition, and health variables, and incorporating more nutritional information on older children, adolescents, and adults into national-level surveys.

### Study limitations

There were 3 main limitations of this study. First, we analyzed matrices of regression results from 218 individual surveys, merged with other data at country- and year-levels. Whereas the geographic and temporal coverage was substantial, the countries and years for which data were available depended on where surveys could be implemented, and did not include many of the world's most vulnerable populations. Second, additional subnational analyses would be valuable, especially because climatic and agricultural risks vary widely within countries. A third limitation was that not all components of the original regression results were used for analysis, namely, the SEs of estimated coefficients on MOB. Instead, we focused on the estimated MOB coefficients themselves. In future work, we would aim to incorporate additional information relating to hypothesis testing, such as the SEs or CIs of estimated MOB coefficients, to gain a deeper understanding of nutrition smoothing and its variability. Finally, we assumed that the errors in mismeasurement of birthdates were random and equally distributed across the year. A more specific approach to dealing with this measurement error might be possible, such as by analyzing recorded birthdates by survey enumerator or calculating an individual child's risk of having a mismeasured birthdate based on observable factors.

### Future research on nutrition smoothing and resilience

Several important questions remain about seasonality in child HAZ and nutrition smoothing. Estimating the amount of stunting that could be eliminated by the smoothing of HAZ outcomes throughout the year could be useful, as well as examining what economic, environmental, and social factors facilitate nutrition smoothing at the national and subnational levels. For example, public health infrastructure and market access might allow families to overcome seasonal environmental risks to their children's health (26). Building on work on gender bias in the intrahousehold allocation of foods, researchers could estimate differences between boys and girls in the smoothing of their HAZ outcomes throughout the year.

Some research questions about nutrition smoothing can be answered using existing data and literature or by developing merged databases that combine different types of data, whereas others could require specialized data collection. For example, including more nutritional outcome data on older children and adolescents would be valuable, especially given that their anthropometric measurements would be less sensitive to artifactual measurement error in MOB. Existing data, information, and knowledge are also valuable. For example, systematic reviews of existing literature on early-life shocks within local contexts would be valuable for synthesizing what is already known about nutrition smoothing in particular places. Making it easier for researchers, especially those in low- and middle-income countries, to study the effects of early-life shocks in their own communities would be productive (27).

Investigating the mechanisms for how early-life shocks affect later health is becoming more feasible due to advances in measurement and in database management for climate and nutrition variables. For example, remote-sensing data have become particularly valuable for obtaining objective information about climatic conditions at particular times (28, 29), or for assessing the risk or severity of famine or drought. Using remote-sensing data does not come without challenges. Remotely sensed climate databases are subject to various biases depending on the particular data-generating processes, but judicious care and various strategies can assess database quality for particular research questions, or address shortcomings during analysis.

Other relatively recent advances of interest to researchers who primarily use publicly available data are the Living Standard Measurement Study-Integrated Surveys on Agriculture, which concurrently measure agricultural and health microdata in nationally representative panels in close collaboration with national government ministries (30). The Demographic and Health Surveys, which collect data in nationally representative repeat cross-sections about every 5 y in low- and middle-income countries, are now including spatial covariate datasets with their geocoded microdata (22). Improving survey enumeration and birth registration efforts to increase the quality of data on MOB would make true seasonal patterns more easily apparent. With the use of these publicly available datasets, there are opportunities for researchers to investigate nutrition smoothing and its local determinants. Making it easier to merge national surveys or censuses with environmental or climate data would also be useful.

There is still no unified understanding of the consequences of seasonal risks to child nutrition. The child health effects of climate and other outside factors are often substantial, and not often homogeneous within countries or across different subgroups of the population in question. Research studies in this area do not often investigate mechanisms directly, largely due to challenges with needed data. Instead, the focus has generally been on measuring the effects on nutrition of early-life shocks or seasonal cycles within specific contexts. These are useful exercises, especially given the heterogeneity in effects described above, and future work should attempt to go deeper in examining mechanisms and biological and social pathways.

## Supplementary Material

nqz111_Supplement_Figure_1

## Notes

This project was supported by a consultancy through The World Bank—Poverty and Equity Global Practice. WAM received additional support for this work from the Bill & Melinda Gates Foundation through the ARENA project (OPP1112307), and the Feed the Future Innovation Lab for Nutrition (AID-OAA-L-10-00006). No funder had a role in the research itself.

Supplemental Figure 1 is available from the “Supplementary data” link in the online posting of the article and from the same link in the online table of contents at https://academic.oup.com/ajcn/.

Abbreviations used: DHS, Demographic and Health Surveys; GDP, Gross Domestic Product; HAZ, height-for-age z-score; MOB, month of birth; OLS, ordinary least squares; PPP, Purchasing Power Parity.
