# Supplementary material for: Correcting for artifactual correlation between misreported month of birth and attained height-for-age reduces but does not eliminate measured vulnerability to season of birth in poorer countries
Source: Am J Clin Nutr. 2019 Jun 10;110(2):485–97. doi: 10.1093/ajcn/nqz111 (PMC6669063; doi:10.1093/ajcn/nqz111)
Supplement: nqz111_Supplement_Figure_1 [file nqz111_supplement_figure_1.docx]

Supplemental Figure 1

Participant flow chart for Finaret, A.B. & W.A. Masters: “**Correcting for artifactual correlation between misreported month of birth and attained height-for-age reduces but does not eliminate measured vulnerability to season of birth in poorer countries**

# ”

**9,747,741 children** total included in this collection

***Stage 1: 218 DHS Surveys***

***“Standard DHS”***

***Stage 2: 218 sets of estimated coefficients of HAZ on MOB***

**8,383,935** without anthropometric measurements

***203 country-year combinations with GDP data to estimate the effect of national incomes on seasonality in HAZ***

**1,363,806 children across 218 surveys** with anthropometric measurements to estimate the effects of MOB on HAZ. Cluster-robust standard errors were used in these estimates (1,2).

References

1. Rogers, W.H. 1993. Regression standard errors in clustered samples. Stata Technical Bulletin 13:19-23. Reprinted in Stata Technical Bulletin Reprints, Vol. 3, 88-94. Available at: <https://www.stata.com/support/faqs/statistics/stb13_rogers.pdf>. Accessed on 24 April 2019.
2. Williams, R.L., 2000. A note on robust variance estimation for cluster‐correlated data. *Biometrics*, *56*(2), pp.645-646.
